# Hospital Meetings

**Published:** 1896-09-05

**Authors:** 


### HOSPITAL MEETINGS.

### MIDDLESEX HOSPITAL.

A special meeting of the governors of the Middlesex Hospital was held on Thursday, August 27th, to consider and approve a scheme for the amalgamation of the medical school with the hospital. August is not a time to expect full meetings, but nevertheless there was a very fair attendance of governors and committee men. Mr. Custance took the chair, and there were also present, amongst others, Sir Ralph Thompson, K.C.B. (chairman of the weekly board), Mr. Francis Hoare, Mr. G. T. Majoribanks (treasurers), Sir Arthur Watson, Mr. Burroughes, Dr. Cayley, Dr. S. Coupland, Dr. Fowler, Dr. W. Pasteur, Mr. L. Hudson, and Dr. A. Voelcker. After the usual *précis* of the transactions of the weekly board had been read, Sir Ralph Thompson briefly detailed the principal features of the new scheme, which has been under consideration for more than two years past. It provides for "the taking over by the hospital of all the property of the school, together with the existing liabilities, and the consequent remission of the debt now due from the school to the hospital. In the future the hospital will receive the gross income of the school, and after payment of necessary charges will provide, according to the profits obtained, a fixed annual sum for the remuneration of the teaching staff," these arrangements to be subject to a revision, if need be, at the end of three years. These changes will involve the constitution of a council of the medical school, composed of the chairman of the weekly board, representatives of the governors, hon. medical officers of the hospital, and the lecturers of the school, who will control all matters relating to the finance and management of the school under the supervision of the board. Sir Ralph Thompson explained that the number of students having lately considerably decreased, it had been felt that some remedy must be applied. The school and the hospital were dependent upon one another, and their amalgamation on the present lines seemed to be the best method of securing their future well-being. Dr. Sydney Coupland gave his cordial approval to the proposed changes, which, in his opinion, would be to the advantage alike of the hospital, the school, and the public. The unanimous consent of all present being signified, and the necessary alteration of the existing laws made, the scheme was adopted, and will duly take effect at the commencement of the new session on October 1st next. Dr. William Pasteur has been appointed dean of the school, vice Dr. Coupland, resigned. The Earl of Derby was elected a vice-president of the hospital.

